# Impact of Histidine–Tryptophan–Ketoglutarate Cardioplegia on Perioperative Prognosis in Surgery Patients for Combined Valvular and Coronary Heart Disease: A Retrospective Study

**DOI:** 10.31083/RCM39546

**Published:** 2025-09-19

**Authors:** Pengrui Si, Haokai Qin, Xunxun Feng, Kun Hua, Xiubin Yang, Mingyang Zhou

**Affiliations:** ^1^Department of Cardiovascular Surgery, Beijing Anzhen Hospital, Capital Medical University, 100029 Beijing, China; ^2^Department of Cardiology, Beijing Anzhen Hospital, Capital Medical University, 100029 Beijing, China; ^3^Department of Medicine, Division of Cardiology, University of California, Los Angeles, CA 90095, USA

**Keywords:** HTK cardioplegia, cold blood cardioplegia, postoperative outcomes, valve surgery, coronary artery bypass grafting

## Abstract

**Background:**

Combined valve and coronary surgery is technically complex, and the prognosis for such patients remains poor. This study aimed to analyze the short-term prognostic effects of histidine–tryptophan–ketoglutarate (HTK) cardioplegia versus 1:4 cold blood (CB) cardioplegia in patients requiring combined valve and coronary surgery.

**Methods:**

This retrospective cohort study categorized patients undergoing valve surgery combined with coronary artery bypass grafting (CABG) into two groups: the HTK group (n = 504) and the CB group (n = 188), based on the type of cardioplegia used. Propensity score matching (PSM) was employed to adjust for baseline differences between the groups. The primary endpoints included operative mortality, postoperative myocardial infarction (PMI), postoperative acute kidney injury (AKI), and postoperative atrial fibrillation (POAF). Secondary endpoints included stroke incidence, ventilation time, aortic cross-clamp time, and intensive care unit (ICU) length of stay (LOS).

**Results:**

After PSM, patients with HTK experienced significantly lower rates of AKI and POAF (*p < *0.05). Troponin I (TnI) and creatine kinase-MB (CK-MB) measurements at 48 and 72 hours postoperatively were lower in the HTK group compared with the CB group (*p < *0.05). However, no significant difference in PMI incidence was detected (*p = *0.368). Additionally, the HTK group demonstrated shorter mechanical ventilation times (*p = *0.01) and ICU stays (*p = *0.009).

**Conclusions:**

HTK cardioplegia reduced postoperative ventilation time, ICU LOS, and the incidence of AKI and POAF compared with CB cardioplegia in patients undergoing valve surgery combined with CABG. HTK cardioplegia is effective, safe, and superior to CB cardioplegia in improving short-term outcomes in these patients.

## 1. Introduction

Extracorporeal cardiopulmonary bypass (CPB) is a critical component of cardiac surgery, providing surgeons with a relatively bloodless and motionless operative field. The establishment of CPB relies on using cardioplegia solutions, which protect cardiomyocytes by reducing metabolic demands and preventing electrolyte and pH imbalances. In clinical practice, two commonly used cardioplegia solutions are histidine-tryptophan-ketoglutarate (HTK) solution and 1:4 cold blood (CB) cardioplegia. HTK is an intracellular, crystalloid cardioplegia solution characterized by its acidic nature, low sodium concentration, and the inclusion of histidine for buffering, mannitol to reduce myocardial edema, and tryptophan to stabilize cell membranes [1]. This solution provides myocardial protection for up to 180 minutes with a single dose, whereas CB cardioplegia sustains protection for only up to 30 minutes per infusion [2]. CB cardioplegia employs a 4:1 dilution ratio, concurrently reducing blood viscosity at hypothermic temperatures to optimize microvascular perfusion while preserving basal oxygen-carrying capacity sufficient to meet diminished metabolic demands during cardiac arrest. Numerous studies have compared the efficacy of HTK and CB cardioplegia in various cardiac procedures. However, limited research exists on their specific use in valve surgery combined with coronary artery bypass grafting (CABG). This combined procedure typically requires longer operative and perfusion times than isolated CABG or valve surgery. Given the significant difference in the number of cardioplegia infusions required when using HTK versus CB during these extended surgeries, we aimed to investigate whether the choice of cardioplegia solution impacts short-term outcomes in this specific surgical context.

## 2. Patients and Methods

### 2.1 Study Populations

**Fig. 1.**
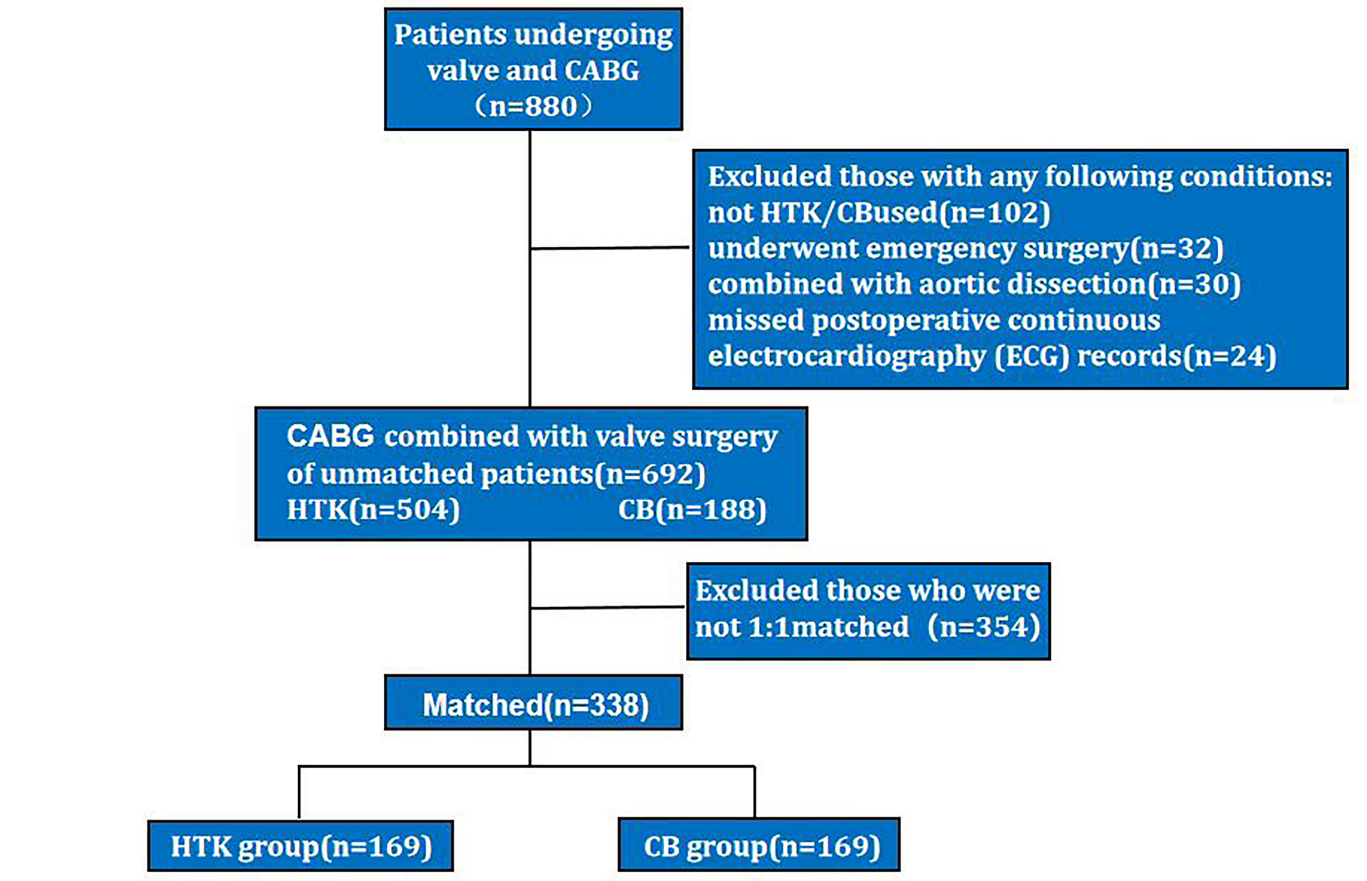
**The flowchart is presented as follows: A total of 880 patients underwent valve surgery combined with coronary artery bypass grafting**. Of these, 188 patients were excluded for not meeting the inclusion criteria. The remaining patients were divided into 504 in the HTK group and 188 in the CB group, based on the type of cardioplegic solution used. After propensity score matching, 169 patients remained in each group. HTK, Histidine-tryptophan-ketoglutarate; CB, cold blood; CABG, coronary artery bypass grafting.

We retrospectively enrolled 880 patients who underwent valve surgery combined with CABG at Beijing Anzhen Hospital between December 2020 and January 2024. The exclusion criteria were as follows: (1) use of cardioplegia solutions other than HTK or CB; (2) concomitant aortic dissection; (3) incomplete clinical data; and (4) emergency surgery. After applying these criteria, 692 patients were included in the study. Of these, 504 patients (73%) received HTK cardioplegia solution (HTK group), and 188 patients (27%) received CB cardioplegia solution (CB group) (Fig. 1).

### 2.2 Surgical Technique

After a median sternotomy, systemic anticoagulation was achieved with 300 to 400 IU/kg of standard heparin. While the pericardium was incised, the graft was prepared for backup. CPB was initiated once the activated clotting time (ACT) exceeded 300 seconds, and systemic hypothermia was maintained at 32 °C to 34 °C. The ascending aorta was cross-clamped, and HTK or CB cardioplegia solution was administered. Cardioplegia solution was delivered via antegrade and retrograde perfusion. The solution was infused through perfusion needles placed in the ascending aorta proximal to the aortic cross-clamp for antegrade perfusion. For retrograde perfusion, the solution was administered through the coronary sinus. Graft perfusion was performed by anastomosing the graft to the distal end and slowly perfusing the cardioplegia solution from the proximal end. Both types of cardioplegia solution were perfused at 4 °C to 8 °C. The composition and usage details of the cardioplegia solution are shown in **Supplementary Table 1**. After cardiac arrest was achieved, CABG was performed first, followed by valve surgery. Once rewarming and adequate venting were completed, the ascending aorta was unclamped, and the heart resumed beating spontaneously. CPB was discontinued after stabilization of the heart rate and blood pressure. The anesthesiologist administered protamine to reverse anticoagulation, with the target ACT maintained below 140 seconds. Hematocrit was kept above 30%.

### 2.3 Data Collection and Diagnostic Criteria

We recorded the patients’ basic preoperative characteristics, medication history, and postoperative complications. Operative mortality was defined as any death, regardless of cause, occurring within 30 days after surgery, either in or out of the hospital [3]. Postoperative acute kidney injury (AKI) is an increase in serum creatinine of 0.3 mg/dL or more within 48 hours from baseline or a urine output of less than 0.5 mL/kg/h for 6 to 12 hours [4]. Perioperative myocardial infarction (PMI) is an elevation of troponin I (TnI) values above the 99th percentile upper reference limit, accompanied by imaging evidence of myocardial ischemia and clinical symptoms. Postoperative atrial fibrillation (POAF) is defined as atrial fibrillation lasting longer than 1 hour and/or requiring treatment after surgery [5]. Postoperative stroke is defined as a permanent neurologic impairment diagnosed by a neurologist and confirmed by imaging evidence of cerebral artery occlusion [6].

### 2.4 Statistical Analysis

The *Kolmogorov-Smirnov test* was used to assess the normality of continuous variables. Continuous variables with a normal distribution were expressed as the mean and standard deviation (SD), and group comparisons were performed using the independent samples *t*-test. Non-normally distributed continuous variables were presented as *interquartile ranges* (IQRs), and intergroup comparisons were performed using the Mann-Whitney U test. Missing values were handled using mean interpolation. Categorical data were analyzed using χ^2^ or *Fisher’s Exact testing.* Fisher’s Exact testing was used to analyze events with less than five expected outcomes. Repeated measures *analysis of variance* (*ANOVA*) was used for repeated variables. Propensity score matching (PSM) was employed to address selection bias. PSM was conducted using the 1:1 nearest-neighbor matching method with a caliper width of 0.2 standard deviations. The PSM analysis included preoperative variables with a *standardized mean difference* (SMD) >0.1 as covariates. These covariates included triple valve surgery, single valve surgery, graft number, white blood cell count (WBC), ratio early to late diastolic transmitral flow velocity (E/A ratio), left internal mammary artery (LIMA) usage, left ventricular ejection fraction (LVEF), creatinine (Cr), hypertension, total cholesterol (TC), TnI, estimated glomerular filtration rate (eGFR), gender, diabetes, and heart failure. After PSM, logistic regression was used to estimate odds ratios (ORs) and 95% confidence intervals (CIs) to evaluate the independent association between the HTK solution group and perioperative outcomes. Common risk factors for mortality associated with cardiac surgery—including age, sex, hypertension, diabetes, body mass index (BMI), LVEF, and TnI—were included in univariate and multivariate logistic regression analyses.

Statistical analyses were performed with R software (version 4.4.1, R Foundation for Statistical Computing). The statistical significance level was set at two-tailed *p* < 0.05.

## 3. Results

### 3.1 Baseline Characteristics 

**Fig. 2.**
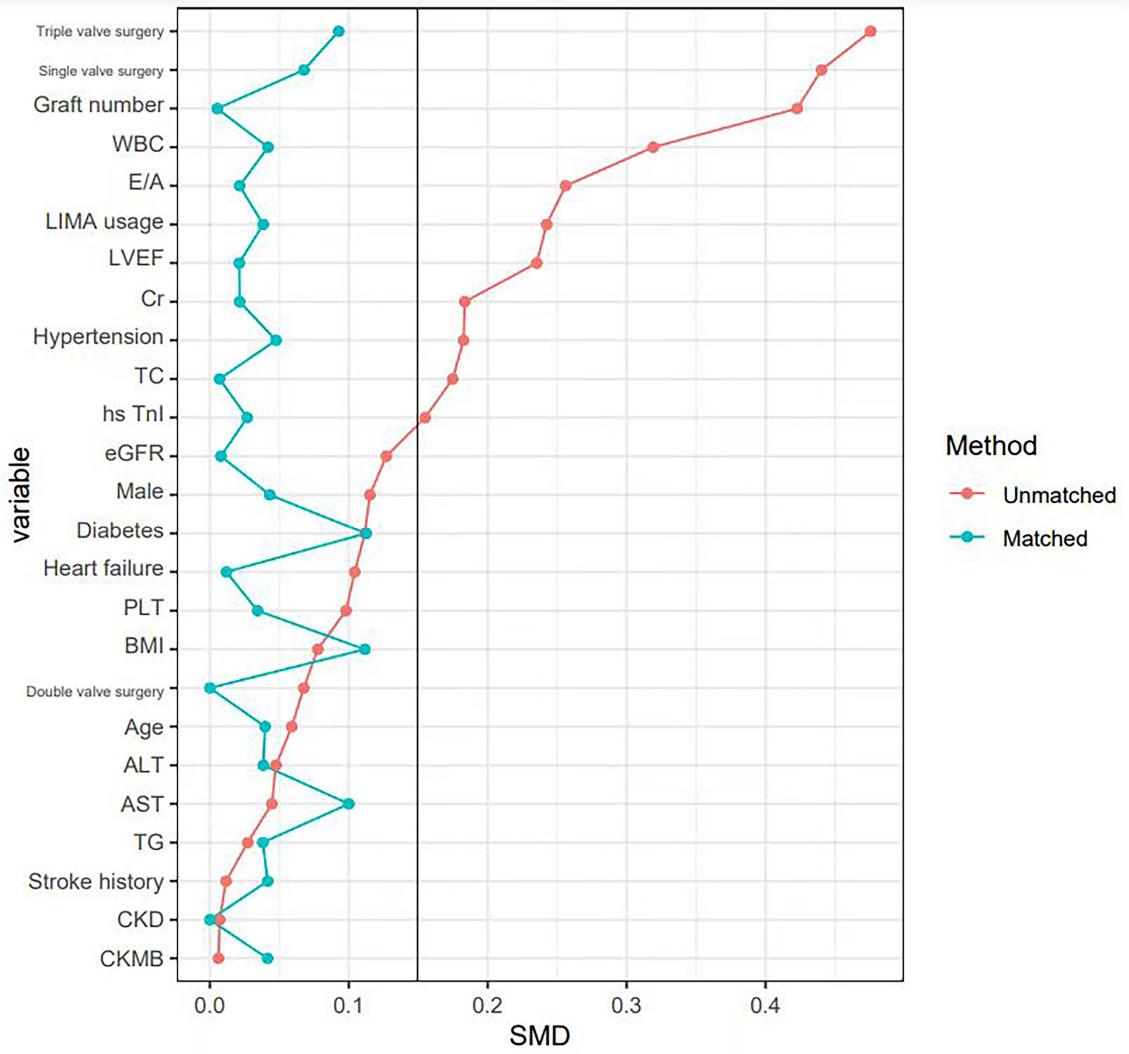
**Covariate balance plot for assessing balance between HTK group and CB group after PSM**. PSM, propensity score matching; WBC, white blood cell count; E/A ratio, ratio early to late diastolic transmitral flow velocity; LIMA, left internal mammary artery; LVEF, left ventricular ejection fractions; Cr, creatinine; TC, total cholesterol; TnI, Troponin I; eGFR, estimated glomerular filtration rate; PLT, platelet; BMI, body mass index; ALT, alanine aminotransferase; AST aspartate transaminase; TG, triglyceride; CKD, chronic kidney disease; CK-MB, creatine kinase-MB; SMD, standardized mean difference.

The preoperative characteristics of patients in each group are presented in Table 1. Significant differences were observed between the HTK group and the CB group regarding history of hypertension, preoperative TC, preoperative WBC count, LVEF, E/A ratio, use of the LIMA, number of grafts, and the proportions of single-valve and triple-valve surgeries (*p* < 0.05). After PSM, differences in baseline data were significantly controlled (SMD <0.15) (Fig. 2).

**Table 1.** **Baseline characteristics between HTK cardioplegia group and cold blood cardioplegia group**.

		Unmatch			PSM		
		HTK (n = 504)	CB (n = 188)	*p* value	HTK (n = 169)	CB (n = 169)	*p* value
Demographics							
	Age, years	64 (57, 68)	64 (57, 68)	0.453	64 (57, 67)	64 (57, 68)	0.679
	Male (%)	375 (74.4)	149 (79.3)	0.224	131 (77.5)	134 (79.3)	0.791
	BMI, kg/m^2^	25.18 (23.12, 27.06)	25.18 (23.36, 26.58)	0.881	25.18 (23.59, 27.38)	25.18 (23.38, 26.64)	0.463
Comorbidity							
	Hypertension (%)	233 (46.2)	104 (55.3)	0.041	95 (56.2)	91 (53.8)	0.743
	Diabetes (%)	105 (20.8)	48 (25.5)	0.222	35 (20.7)	43 (25.4)	0.366
	CKD (%)	5 (1.0)	2 (1.1)	1.000	1 (0.6)	1 (0.6)	1.000
	Stroke history (%)	50 (9.9)	18 (9.6)	1.000	14 (8.3)	16 (9.5)	0.848
	Heart failure (%)	258 (51.2)	106 (56.4)	0.258	93 (55)	94 (55.6)	1.000
Preoperative laboratory data							
	TG, mmol/L	1.40 (1.02, 1.72)	1.43 (1.09, 1.76)	0.475	1.44 (1.04, 1.78)	1.50 (1.11, 1.81)	0.508
	TC, mmol/L	4.17 (3.57, 4.87)	4.15 (3.44, 4.47)	0.042	4.17 (3.36, 4.63)	4.17 (3.46, 4.46)	0.851
	Cr, µmol/L	79.9 (69.97, 94.23)	81.3 (70.35, 96.73)	0.509	77.3 (67.8, 95.5)	80.4 (69.9, 94.1)	0.858
	ALT, U/L	17 (12.00, 26.00)	18 (12.00, 29.00)	0.166	17 (12, 30)	18 (12, 28)	0.789
	AST, U/L	20 (15.00, 32.00)	21 (17.00, 35.00)	0.088	21 (16, 42)	21 (17, 34)	0.836
	PLT, 10^9^/L	160.5 (110.00, 207.00)	149 (106.75, 199.25)	0.264	158 (103, 211)	149 (110, 198)	0.861
	WBC, 10^9^/L	7.46 (5.93, 9.89)	8.5 (5.99, 12.31)	0.003	8.42 (6.41, 11.95)	8.32 (5.93, 11.66)	0.444
	eGFR, mL/min	85.4 (69.51, 95.90)	83.47 (67.20, 96.36)	0.433	86.01 (69.02, 97.85)	84.64 (68.51, 97.2)	0.856
	CK-MB, ng/mL	2 (1.40, 3.20)	2.05 (1.30, 3.32)	0.975	2.2 (1.5, 3.9)	2 (1.3, 3.2)	0.207
	TnI, ng/mL	2.3 (1.3, 3)	2 (1.17, 2.73)	0.104	2 (1.1, 2.73)	2 (1.2, 2.73)	0.675
Preoperative echocardiographic data							
	LVEF, %	58 (52, 63)	55 (48, 60.25)	0.005	56 (50, 63)	56 (50, 61)	0.684
	E/A ratio	1.24 (0.79, 1.47)	0.94 (0.67, 1.33)	0.001	0.94 (0.74, 1.28)	0.94 (0.68, 1.34)	0.553
CABG data							
	LIMA usage (%)	112 (22.2)	62 (33)	0.005	51 (30.2)	54 (32.0)	0.814
	Graft number	2 (1, 3)	2 (1, 3)	0.001	2 (1, 3)	2 (1, 3)	0.991
Types of valve surgery							
	Single valve surgery	290 (57.5)	146 (77.7)	0.001	123 (72.8)	128 (75.7)	0.619
	Double valve surgery	79 (15.7)	25 (13.3)	0.514	24 (14.2)	24 (14.2)	1.000
	Triple valve surgery	135 (26.8)	17 (9.0)	0.001	22 (13.0)	17 (10.1)	0.496

Data are presented as median [25th–75th percentiles] or n (%). BMI, body mass index; CKD, chronic kidney disease; TG, triglyceride; TC, total cholesterol; Cr, creatinine; ALT, alanine aminotransferase; AST aspartate transaminase; PLT, platelet; WBC, white blood cell; eGFR, estimated glomerular filtration rate; CK-MB, creatine kinase MB; TnI, Troponin I; LIMA, left internal mammary artery; LVEF, left ventricular ejection fractions; E/A, ratio early to late diastolic transmitral flow velocity.

### 3.2 Postoperative Outcomes 

**Fig. 3.**
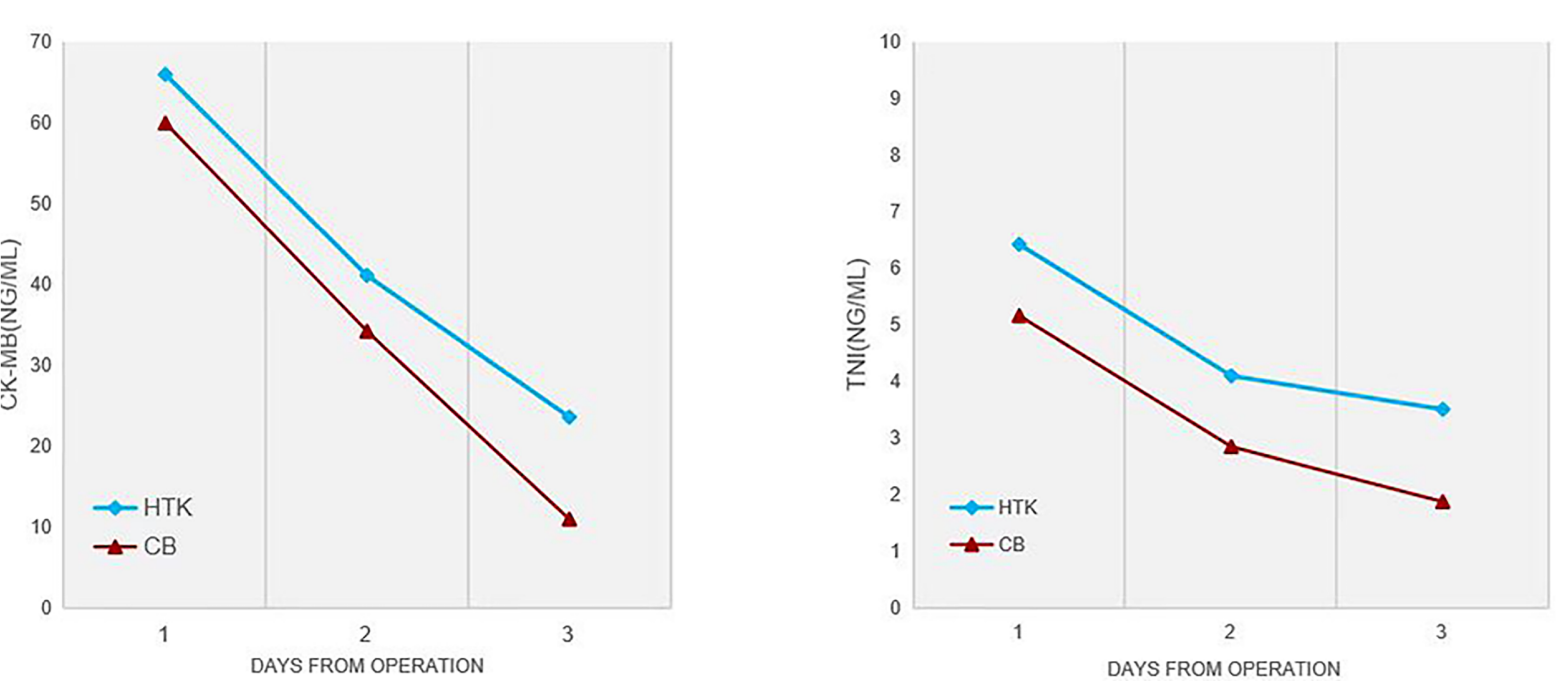
**The researchers measured TnI and CK-MB concentrations in both groups at 24, 48, and 72 hours after surgery**. The results are reported as medians.

A total of 27 patients in both groups experienced operative mortality, and the difference between the HTK and CB groups was not statistically significant after PSM (3.6% vs 5.3%, *p* = 0.597). The incidence of AKI (7.7% vs. 18.3%, *p* = 0.006) and POAF (36.7% vs. 47.9%, *p* = 0.048) was significantly lower in the HTK group. Serial measurements of TnI and CK-MB were significantly higher in the HTK group compared to the CB group at 48 and 72 hours postoperatively (*p* < 0.05) (Fig. 3). However, there was no statistically significant difference in the incidence of PMI between the two groups. The HTK group had a shorter duration of mechanical ventilation (*p =* 0.01) and ICU stay (*p* = 0.009). No significant differences were observed in CPB time (*p* = 0.366) or cross-clamp time (*p* = 0.552). The remaining postoperative outcomes did not differ significantly between the two groups (Table 2).

**Table 2.** **Postoperative outcomes between HTK cardioplegia group and cold blood cardioplegia group after PSM**.

		HTK (n = 169)	Cold blood (n = 169)	*p* value
Operative mortality (%)		6 (3.6)	9 (5.3)	0.597
POAF (%)		62 (36.7)	81 (47.9)	0.048
Stroke (%)		4 (2.4)	5 (3.0)	1.000
AKI (%)		13 (7.7)	31 (18.3)	0.006
PMI (%)		4 (2.4)	1 (0.6)	0.368
CK-MB, ng/mL				
	24 h	66 (42, 96)	60 (34, 104)	0.353
	48 h	41.1 (25, 76)	34.2 (19.1, 62.4)	0.005
	72 h	23.6 (9.9, 48.3)	11 (6.2, 20.5)	0.001
TnI, ng/mL				
	24 h	6.42 (3.56, 10.95)	5.16 (2.45, 8.67)	0.132
	48 h	4.10 (2.12, 7.74)	2.85 (1.46, 5.73)	0.002
	72 h	3.51 (1.26, 3.75)	1.88 (0.91, 3.51)	0.003
Operation time, hours		5.5 (5, 7)	6 (5, 7)	0.252
Crossclamp time, min		111 (94, 144)	112 (96, 135)	0.552
CPB time, min		173 (145, 211)	168 (147, 200)	0.366
Ventilation time, hours		24.5 (18.0, 50.5)	38.5 (21.0, 72.5)	0.009
LOS, days		8 (6, 10)	8 (6, 11)	0.701
ICU time of stay, hours		37 (19, 68)	47 (23, 95)	0.009
IABP (%)		19 (11.2)	21 (12.4)	0.866
ECMO (%)		4 (2.4)	2 (1.2)	0.679

Data are presented as median [25th–75th percentiles] or n (%). POAF, postoperative atrial fibrillation; AKI, acute kidney injury; PMI, postoperative myocardial infarction; CK-MB, creatine kinase MB; TnI, Troponin I; CPB, cardiopulmonary bypass; LOS, length of stay; ICU, intensive care unit; IABP, intra-aortic ballon pump; ECMO, extracorporeal membrane oxygenator.

Logistic univariate regression analysis revealed a significant association between the intraoperative use of HTK cardioplegia and a reduced risk of AKI (OR, 0.37; 95% CI, 0.18–0.72; *p* = 0.005) and POAF (OR, 0.63; 95% CI, 0.41–0.97; *p* = 0.037). These associations were further confirmed in multivariate logistic regression analyses (**Supplementary Table 2**).

### 3.3 Subgroup Analysis

**Fig. 4.**
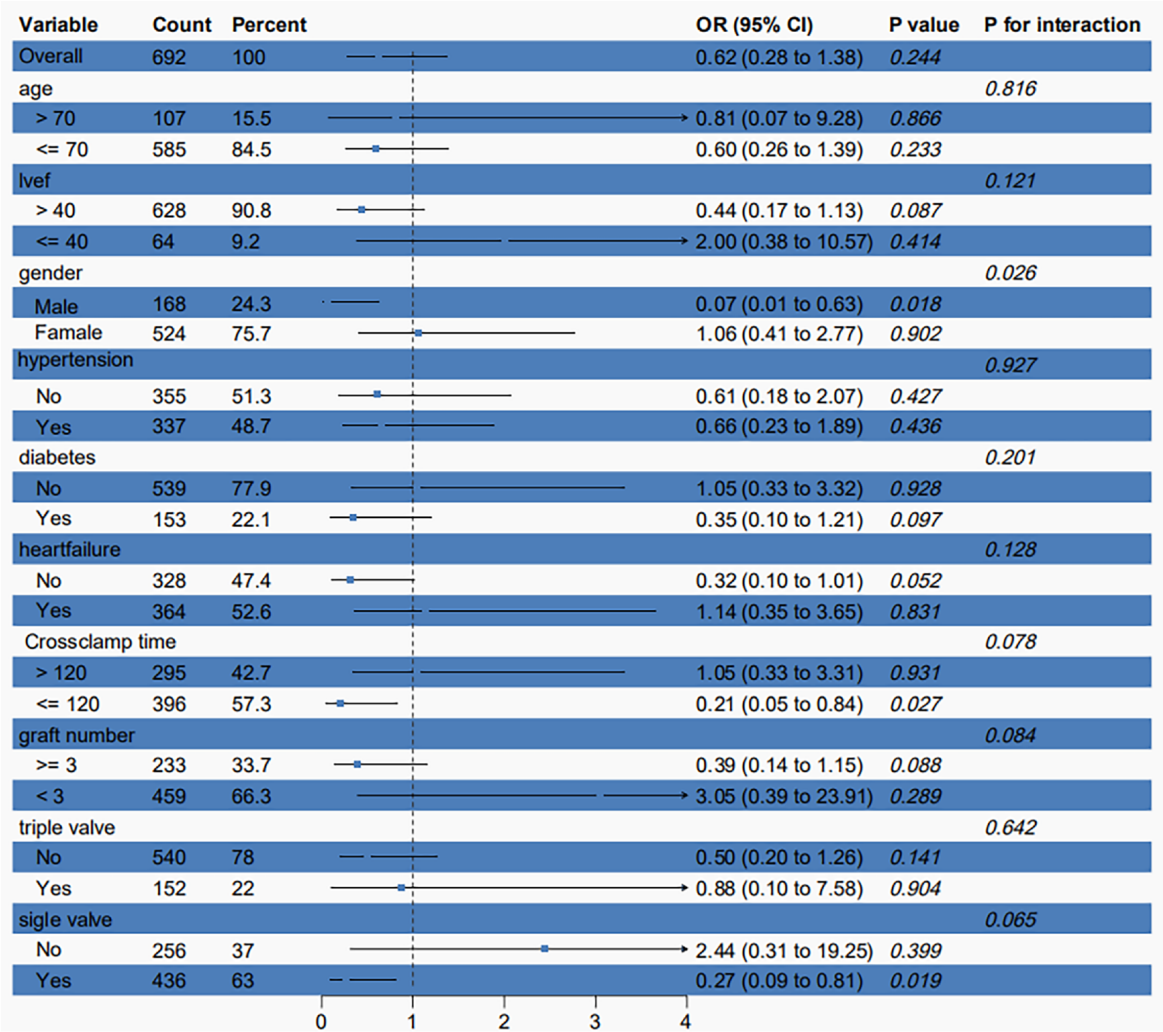
**Difference in the incidence of operative mortality according to major subgroups of interest**.

Subgroup analyses stratified by age, LVEF, gender, hypertension, diabetes, heart failure, cross-clamp time, graft number, single-valve surgery, and double-valve surgery were performed to compare the HTK group and the CB group. The use of HTK cardioplegia was associated with lower operative mortality in female patients during single-valve surgery and when the cross-clamp time exceeded 120 minutes. No significant association between the use of HTK cardioplegia and operative mortality was observed in the other subgroups (Fig. 4).

## 4. Discussion

HTK cardioplegia solution, developed by Bretschneider in the 1970s, has been utilized as a cardioprotective agent in various types of cardiac surgery [7]. Studies have indicated that a single infusion of HTK can provide myocardial protection for up to 180 minutes; however, its empirical usage duration is often limited to 120 minutes [8]. HTK has been employed for many years, and numerous studies have demonstrated its reliability across different cardiac surgical procedures. Nevertheless, many studies adequately compare HTK with CB cardioplegia in valve surgery combined with CABG. Previous findings on HTK and CB in various cardiac procedures have generally been neutral. In a retrospective study, Viana *et al*. [7] reported no significant difference in operative mortality between the HTK and CB strategies in patients undergoing complex cardiac surgery. In a prospective randomized trial, Ak *et al*. [9] found no significant differences between the two groups in rates of postoperative intra-aortic balloon pump use, POAF, or neurological complications. Tshomba *et al*. [10] compared HTK and Ringer’s solution as renal perfusion fluids in thoracoabdominal aortic aneurysm repair and found that HTK reduced the incidence of AKI.

The efficacy of HTK solution in myocardial protection and recovery of cardiac function for patients undergoing cardiac surgery remains controversial. For instance, one study reported that HTK provided myocardial protection comparable to traditional CB cardioplegia [11, 12]. Another study found that the CB group had a lower incidence of PMI and demonstrated better protection of cardiac function than the HTK group [13]. Our study demonstrated that TnI and CK-MB measurements at 48 and 72 hours postoperatively were higher in the HTK group compared with the CB group. However, the two groups had no significant difference in the incidence of PMI or operative mortality. We believe that, despite the elevated levels of CK-MB and TnI observed in the HTK group during the postoperative period, these values remained within the normal reference range and were well below the thresholds used to define PMI [14]. This interpretation is supported by a study conducted by Devereaux *et al*. [15], which found that TnI levels associated with an increased risk of operative mortality after cardiac surgery were significantly higher than the upper limit of the current clinical reference values used to define perioperative myocardial injury. The myocardial protective effect of HTK in valve surgery combined with CABG is comparable to that of CB, a finding that can be attributed to the unique mechanism of HTK. HTK is an intracellular, pure crystalline cardioplegia solution free of blood components. It has a low calcium content and includes the calcium ion blocker magnesium sulfate, effectively reducing calcium overload during ischemia-reperfusion injury. Additionally, HTK is more likely to be distributed uniformly in the cardiac microvasculature at low temperatures due to its lack of blood components. This property particularly benefits patients with concomitant coronary artery disease [16].

AKI is a common complication following cardiac surgery and significantly affects patient prognosis [17, 18]. Valve surgery combined with CABG is technically complex, involves prolonged operative times, and is more likely to result in postoperative kidney injury. Patients with renal failure requiring hemodialysis are at an increased risk for acute respiratory failure, cardiac insufficiency, and systemic circulatory disturbances after surgery [19]. HTK has been used for many years as a cardioprotective solution with well-established efficacy, and it has subsequently become a preservation solution for various organs in transplantation, particularly playing a critical role in kidney transplantation. A meta-analysis of kidney transplants by O’Callaghan *et al*. [20] demonstrated a lower incidence of delayed graft function recovery with HTK than other preservation solutions. HTK has also been widely used to prevent renal injury associated with major vascular surgery. In a randomized thoracic and abdominal aortic aneurysm repair study, HTK used as a renal perfusate demonstrated superior renal protection compared to CB perfusate [21]. In our study, we observed the beneficial effects of HTK in reducing the incidence of AKI. Cardioplegia solutions enter the kidneys and other organs through the bloodstream during cardiac surgery. Compared to extracellular solutions such as CB cardioplegia, HTK reduces cellular edema and ischemic damage. This effect is attributed to HTK’s high histidine content, which provides buffering capacity, reduces inflammation and oxidative stress, and mitigates acidosis associated with anaerobic metabolism. One of the advantages of using HTK is that it eliminates the need for frequent interruptions during surgical administration. Frequent administration of pressurized cardioplegia solutions through the aortic root can increase the risk of atherosclerotic plaque dislodgement. Mukdad L *et al*. [22] demonstrated a lower incidence of microembolism with a single dose of cardioplegia solution. These microemboli can contribute to AKI by occluding renal blood vessels through the bloodstream [22]. However, some studies have concluded that there is no statistically significant difference between HTK and CB cardioplegia solutions regarding postoperative cardiac surgery-associated AKI incidence. This discrepancy may be attributed to variations in the type of surgery, and further studies are needed to validate these findings.

POAF is caused by conduction disturbances resulting from insufficient intraoperative myocardial protection and is associated with mechanical injury, oxidative stress, and non-uniform perfusion [11]. A network meta-analysis demonstrated a significantly lower risk of POAF in the HTK group than in the CB group. We hypothesize that the reduction in POAF with HTK is attributable to its high histidine content, which maintains anaerobic glycolysis and inhibits inflammatory factors through nitric oxide (NO) production. This mechanism allows the atrial muscle to consume less oxygen while enhancing its recovery rate of high-energy phosphates and improving contractile efficacy, ultimately reducing the incidence of atrial fibrillation [23].

We observed that HTK reduced operative mortality when aortic cross-clamp time exceeded 120 minutes. Given that a single infusion of HTK provides myocardial protection for up to 180 minutes, it is particularly advantageous for procedures with cross-clamp times >120 minutes. A single infusion of HTK can meet the surgical requirements while ensuring safety and avoiding the need for frequent infusions, as required with CB cardioplegia solutions. This minimizes interruptions and ensures surgical continuity. In addition, single-valve procedures demonstrated significant benefits with HTK, including shorter CPB times, reduced myocardial damage, and lower mortality rates. In the HTK group, female patients had a lower mortality rate than male patients. This difference may be related to the higher BMI of male patients and the influence of androgens, which increase platelet aggregation in the microcirculation, leading to microthrombosis and an elevated risk of renal and cerebral complications [24].

## 5. Limitation

Our study has several limitations. First, it was a single-center retrospective study. Although we used PSM to minimize potential bias, we could not eliminate the influence of unknown confounders. Second, we were unable to collect all intraoperative and postoperative variables. For example, the study design did not include the number of intraoperative cardioplegia solution infusions, the method of cardioplegia solution administration, and postoperative EF, which may have introduced bias. Finally, as this was a retrospective study, we could not evaluate the impact of HTK cardioplegia solution on long-term prognosis.

## 6. Conclusion

In valve surgery combined with CABG, HTK cardioplegia solution is a safe and reliable alternative to CB cardioplegia. Compared with CB cardioplegia, HTK cardioplegia reduces postoperative ventilation time and ICU length of stay and demonstrates significant advantages in reducing the incidence of POAF and AKI. These benefits make HTK cardioplegia an effective option for adult patients undergoing valve surgery combined with CABG.

## Availability of Data and Materials

The datasets generated and analyzed during the current study are not publicly available due to the nature of this research, participants of this study did not agree for their data to be shared publicly but are available from the corresponding author on reasonable request.

## Supplementary Material

Supplementary material associated with this article can be found, in the online version, at https://doi.org/10.31083/RCM39546.
