# Supplementary material for: Impact of Histidine–Tryptophan–Ketoglutarate Cardioplegia on Perioperative Prognosis in Surgery Patients for Combined Valvular and Coronary Heart Disease: A Retrospective Study
Source: Rev Cardiovasc Med. 2025 Sep 19;26(9):39546. doi: 10.31083/RCM39546 (PMC12516746; doi:10.31083/RCM39546)
Supplement: Supplementary file 1 [file 2153-8174-26-9-39546-s1.docx]

**Supplementary Table 1. Composition of Cardioplegia Solutions**

|  | **HTK cardioplegia** | **Cold blood cardioplegia** |
| --- | --- | --- |
| **Na+(mmol/L)** | 15 | 140 |
| **K+(mmol/L)** | 9 | 20 |
| **Mg+(mmol/L)** | 4 | 13 |
| **Ca+(mmol/L)** | 0.015 | 0 |
| **Histidine(mmol/L)** | 198 | 0 |
| **Tryptophan(mmol/L)** | 2 | 0 |
| **Ketoglutarate(mmol/L)** | 1 | 0 |
| **Mannito(mmol/L)** | 30 | 0 |
| **PH** | 7.02-7.20 | NA |
| **Temperature(°C)** | 4-8 | 4-8 |
| **Induction dose** | 20-30 ml/kg,  up to 2000 ml | mixing with 3/4 blood,  20 ml/kg, up to 800 ml |
| **Reinfusion interval (minutes)** | 60-120, even more | 20 |
| **Maintenance dose** | 500 ml | 100-200 |

**Supplementary Table 2. The ORs for postoperative outcomes in HTK group after PSM**

| **Methods** | **Univariate analysis** | | **Multivariate analysis** | |
| --- | --- | --- | --- | --- |
|  | OR (95%CI) | p value | OR (95%CI) | p value |
| **POAF** | 0.63 [0.41, 0.97] | 0.037 | 0.64 [0.41, 1.00] | 0.049 |
| **Perioperative mortality** | 0.65 [0.22, 1.86] | 0.431 | 0.58 [0.17, 1.77] | 0.344 |
| **Stroke** | 0.86 [0.40, 1.83] | 0.702 | 0.86 [0.39, 1.85] | 0.692 |
| **AKI** | 0.37 [0.18, 0.72] | 0.005 | 0.35 [0.17, 0.69] | 0.003 |
| **PMI** | 4.07 [0.59, 80.12] | 0.211 | 3.50 [0.42, 76.59] | 0.300 |

POAF, postoperative atrial fibrillation; AKI, acute kidney failure; PMI,

perioperative myocardial infarction; OR, odds ratio; CI, confidence intervals.
